# Combination of the Parasitoid *Spalangia endius* Walker and Chemical Pesticides for the Control of *Zeugodacus cucurbitae* (Coquillett)

**DOI:** 10.3390/insects16070716

**Published:** 2025-07-12

**Authors:** Lei Li, Dongyin Han, Jing Zhao, Haiyan Qiu, Fangping Zhang, Zhengpei Ye, Yueguan Fu

**Affiliations:** 1 Key Laboratory of Integrated Pest Management on Tropical Crops, Ministry of Agriculture and Rural Affairs, Environment and Plant Protection Institute, Chinese Academy of Tropical Sciences, Haikou 571101, China; lee_lay@catas.cn (L.L.);; 2 Sanya Research Institute of Chinese Academy of Tropical Agricultural Sciences, Sanya 572025, China

**Keywords:** melon fly, pupal parasitoid, abamectin, toxicity

## Abstract

*Spalangia endius* Walker, a pupal parasitoid of the alien invasive pest *Zeugodacus cucurbitae* (Coquillett), causes 33% host mortality. This study assessed whether combining *S. endius* with insecticides (abamectin, thiamethoxam, nitenpyram, emamectin benzoate, or beta-cypermethrin)—all effective against *Z. cucurbitae*—could enhance control efficacy. Among these, abamectin was the least toxic to adult *S. endius*. Surface contact treatments with 12 and 15 mg a.i./kg of abamectin did not significantly increase *S. endius* mortality. However, mixing 12 mg a.i./kg of abamectin into a honey solution to encourage ingestion decreased the survival, parasitism, and fecundity of *S. endius*. In olfactometer assays, *S. endius* adults avoided abamectin-treated host pupae, though prior exposure to abamectin mitigated this avoidance. The timing of abamectin soil application relative to host pupation and *S. endius* release affects host mortality. The most effective timing is spraying abamectin before host pupation (to expose *Z. cucurbitae* larvae) and then releasing *S. endius*. Field trials confirmed that combining abamectin (12 mg a.i./kg) with *S. endius* increased host mortality more than either treatment alone. In conclusion, abamectin (12 mg a.i./kg) is a suitable insecticide for combination with *S. endius* to control *Z. cucurbitae.* The application sequence should be spraying abamectin before hosts pupate and, only after that, releasing the parasitoid.

## Simple Summary

The melon fly (*Zeugodacus cucurbitae*) is a pest that prefers to bore into and feed on cucurbitaceous crops and that pupates in the soil. The parasitoid *Spalangia endius* can burrow into the soil to attack melon fly pupae, but it only kills about 33% of them. Since insecticides are essential for controlling melon fly populations, we assessed how five commonly used insecticides affect the toxicity, survival, reproduction, choice-making behaviour, and parasitism ability of *S. endius*. Our goal was to determine how to combine insecticides with *S. endius* to more effectively control melon fly pupae. Through a series of laboratory and semi-field trials, we confirmed that abamectin is suitable for combined use with *S. endius*. However, we must be careful about the concentration and timing of abamectin application. The best approach is to spray abamectin on the soil surface before the melon flies pupate and then release the parasitoids.

## 1. Introduction

The melon fly, *Zeugodacus cucurbitae* (Coquillett) (Diptera: Tephritidae), which originated in Central Asia, has been widely and inadvertently introduced in the temperate, tropical, and subtropical regions of the world [1,2]. It can attack over 120 types of vegetables and fruits, especially fruits from the Cucurbitaceae family [1,3]. The damage caused by *Z. cucurbitae* mainly results from female adults ovipositing in tender tissues of the hosts. Larvae bore into the hosts, causing further damage. It usually results in hosts etiolating, rotting, or developing abnormalities [1].

The utilisation of parasitoids is one important strategy for the control of *Z. cucurbitae*, playing an essential role in the integrated pest management of *Z. cucurbitae* in various regions worldwide [4,5,6,7,8]. *Spalangia endius* Walker (Hymenoptera: Pteromalidae) is an important pupal parasitoid of *Z. cucurbitae* and was the first parasitoid recorded as serving as a major strategy of control for *Z. cucurbitae* in the industry standards of the Ministry of Agriculture and Rural Affairs (MARA) of the People’s Republic of China (PRC) [7,9]. Typically, a female of the species uses her ovipositor to drill the puparium of a host, where she deposits an egg, or she may extract fluids from a host wound. Alternatively, she may leave without depositing an egg, which can lead to dehydration and death of the pupae [7,10]. Research has demonstrated that under field conditions, when the ratio of *S. endius* to *Z. cucurbitae* pupae is maintained at 1:10, the parasitoid can induce a mortality rate of up to 33% in the melon fly pupae [7]. However, the sole utilisation of *S. endius* cannot completely kill the pupae of *Z. cucurbitae* in the field. Other control strategies are needed to enhance the efficacy of *S. endius*.

In addition to the use of parasitoids, many other strategies have been developed to control *Z. cucurbitae*, including trapping, the sterile insect technique, usage of entomopathogenic fungi, etc. [1,11,12,13]. However, to date, the application of insecticides has been the essential control strategy in most of the countries where *Z. cucurbitae* occurs, including China [8,14]. Abamectin, thiamethoxam, nitenpyram, emamectin benzoate, and beta-cypermethrin are recommended and used for controlling *Z. cucurbitae* according to the industry standards of MARA of the PRC [9]. Because *S. endius* is also an important agent for the control of *S. endius* in China, the combined application of insecticides and *S. endius* is particularly important. In the current study, different insecticides with strong control efficacy against *Z. cucurbitae* were selected to evaluate their toxicities against *S. endius* and to develop a strategy for the combined application of the parasitoid and screened insecticides.

## 2. Materials and Methods

### 2.1. Insect Rearing, Insecticides, and Olfactometer

*Spalangia endius* and *Z. cucurbitae* were collected from bitter gourd fields (18°19′ N, 108°50′ E) in Sanya, Hainan, China, and reared in laboratory cultures at 26 ± 1 °C, 65 ± 5% relative humidity (RH), and a photoperiod of L:D = 14:10. The larvae of *Z. cucurbitae* tested in the experiments were reared on an artificial diet consisting of sodium benzoate, methyl 4-hydroxybenzoate, beer yeast powder, sucrose, pumpkin, wheat bran, and water (1:1:25:50:100:100:125 by weight) [7]. The pupae of *Z. cucurbitae* were collected and maintained in sand with 10% water content. Adults of *S. endius* were propagated on pupal *Z. cucurbitae* for more than 25 generations.

In the current study, five standards were selected: 97% abamectin, 95% thiamethoxam, and 99% nitenpyram (all sourced from Shanghai Macklin Biochemical Technology Co., Ltd., Shanghai, China); 98% emamectin benzoate (from Shanghai Yuanye Bio-Technology Co., Ltd., Shanghai, China); and 99% beta-cypermethrin (from Shanghai Aladdin Biochemical Technology Co., Ltd., Shanghai, China). Additionally, a commercial insecticide, 1.8% abamectin microemulsion (supplied by Shenzhen Noposion International Investment Co. Ltd., Shenzhen, China), was also chosen for the study.

The olfactometer was assembled from, in sequence, a Y-shaped tube (length of the main tube, 20 cm; length of each arm, 15 cm; inner diameter, 1.4 cm; and 75° angle between the arms), two flowmeters (model number LZB-3B, Yuyao Yinhuan Flowmeter Co., Ltd., Yuyao, China), two odour source bottles (250 mL), two distilled water bottles (250 mL), two activated charcoal bottles (500 mL), and an air pump. All the above accessories were connected via odourless silicone tubes. A steady airflow of 200 mL/min was directed toward the two arms of the Y-shaped tube through the flowmeter control.

### 2.2. Determination of the Toxicity of Different Insecticides to S. endius

The toxicities of the five different insecticides to *S. endius* were examined using the residual film method [15,16,17]. The abamectin standard was diluted with 99.5% acetone (Guangzhou Chemical Reagent Factory, Guangzhou, China) at concentrations of 10, 20, 40, 60, and 100 mg a.i./L. Similarly, thiamethoxam (10, 20, 50, 100, and 200 mg a.i./L), nitenpyram (1, 5, 10, 20, and 40 mg a.i./L), emamectin benzoate (0.1, 1, 5, 10, and 20 mg a.i./L), and beta-cypermethrin (1, 10, 20, 30, and 40 mg a.i./L) standards were also diluted with acetone. Three millilitres of each diluted solution was pipetted into a glass tube (4.5 cm diameter, 11.5 cm long). Subsequently, the tube was placed horizontally and rolled rapidly to ensure that the inner wall was uniformly coated with the solution, continuing until no droplets were visible on the glass surface. Control tubes were treated similarly after 3 mL of acetone was added. Then, these tubes were left at room temperature (26–28 °C) for 1 h to ensure the complete evaporation of acetone before the tested insects were introduced. A total of 60 two-day-old *S. endius* adults were independently placed in each tube. The tube was covered with a piece of gauze (0.15 mm pore diameter), and a cotton ball soaked in a 10% honey water solution (honey produced by Hainan Zhuojin Fengye Co., Ltd., Haikou, China) was placed in the centre. All treatments and the control were repeated six times. These tubes were transferred to a chamber set under the same environmental conditions as those used for insect rearing. After 24 h, the number of live and dead *S. endius* was counted.

### 2.3. Assessment of the Survival of S. endius Under Varying Concentrations and Deposition Durations of Abamectin, Along with Distinct Test Methods

Following the outcomes of the experiment described in Section 2.2, the residual film and impregnated filter methods were used to evaluate the effect of abamectin on the survival of *S. endius* [18]. The tests were conducted in two types of glass tubes (4.5 cm in diameter, 11.5 cm in height). One was an empty glass tube, and the other was a tube with an equal surface area of filter papers (approximately 178.39 cm^2^), which adhered to the bottom and inner walls. The abamectin standard was diluted with acetone at concentrations of 12, 15, and 18 mg a.i./kg, according to the recommended concentration for the control of *Z. cucurbitae* [9]. Three millilitres of each diluted solution was pipetted into the two types of tubes and rolled rapidly to ensure that the solution uniformly covered the inner wall or filter papers. The two types of tubes treated with only 3 mL of acetone served as controls. After the acetone evaporated for 1 h, the tubes of the treatments and controls were placed into a chamber set under the same environmental conditions as those used for insect rearing. After 0, 2, 4, and 6 days of storage, 60 two-day-old *S. endius* adults were successively placed into the tubes. Then, the tubes were covered with a piece of gauze (0.15 mm pore diameter), and a cotton ball soaked in a 10% honey water solution was placed in the centre. All treatments and controls were repeated four times. The tubes of all treatments and controls were transferred to a chamber with the same environmental conditions as those used for insect rearing. After 24 h, the number of live and dead *S. endius* in each treatment and control were recorded.

### 2.4. Measuring the Survival and Parasitism of S. endius Fed with a Mixture of Abamectin and Honey Solution

All tests were conducted in glass tubes (4.5 cm in diameter, 11.5 cm in height) with an equal surface area of filter papers (approximately 178.39 cm^2^) adhering to the inner wall and bottom. The abamectin standard was diluted in acetone (12 mg a.i./kg). For the survival test of *S. endius*, 3 mL of the diluted solution was pipetted into the tube, which was then laid flat and rolled rapidly to ensure that the solution uniformly covered the filter paper. Control tubes were treated similarly after 3 mL of acetone was added. After the acetone evaporated for 1 h, 3 mL of 10% honey water solution and 0.05 mL of 0.01% Tween 80 (Shanghai Yuanye Bio-Technology Co., Ltd., Shanghai, China) were mixed and added to the filter papers in each tube. The tube was then covered with gauze. Subsequently, 100 two-day-old adult *S. endius* starved for 5 h were placed in each tube. The treatment and control experiments were repeated eight times. These tubes were placed in a chamber set under the same environmental conditions as those used for insect rearing. After 3 h, all *S. endius* from each tube were transferred into a clean glass tube and cultured under the same conditions. Each tube was provided with a cotton ball soaked in 10% honey water solution. The number of live and dead *S. endius* was recorded after 12, 24, 48, 72, and 96 h.

For the parasitic efficacy test of *S. endius*, the operating procedure before *S. endius* was transferred from the treatment and control tubes to clean tubes was the same as that of the survival test in the current study. After 3 h, 10 pairs of live *S. endius* were selected from each treatment and control tube and separately placed in a clean tube containing 100 three-day-old *Z. cucurbitae* pupae. Both the treatment and control were repeated 10 times. The remaining live *S. endius* from each treatment and control were gathered separately and transferred to two plastic boxes (20 cm × 20 cm × 20 cm) for use in replacing the dead *S. endius* in subsequent tests. Into each box, 200 *Z. cucurbitae* pupae were placed in advance. The tubes and boxes were then sealed with gauze; a cotton ball soaked in a 10% honey water solution was placed in the centre, and the boxes were placed in the incubation chamber. Every 24 h, all pupae in each tube and box were replaced with an equal quantity of three-day-old *Z. cucurbitae* pupae using the same honey cotton ball. If any *S. endius* had died in the treatment and control tubes, an equal number of *S. endius* were replenished from the two above-mentioned plastic boxes. The pupae replaced for each treatment and control were transferred into new clean tubes and cultured under the same conditions. After 6 days, the emergence of *Z. cucurbitae* was recorded for three consecutive days. After 20 days, the number of *S. endius* that emerged from *Z. cucurbitae* pupae was counted for five consecutive days. The leftover pupae that did not successfully develop into adult Z. cucurbitae or adult *S. endius* were gathered. These pupae were dissected to quantify the number of pupae that were parasitised but did not successfully emerge as adult *S. endius* [7]. This measure of parasitism ignores hosts that were parasitised but notes whether the *S. endius* offspring died in the egg or early larval stage because whether dead eggs and early larvae were absent or undetectable upon dissection cannot be distinguished.

### 2.5. Determination of the Olfactory Behavioural Responses of S. endius to Abamectin

To clarify whether the odour of abamectin influences the selection of *S. endius* to *Z. cucurbitae* pupae, the behavioural responses of *S. endius* were examined using a Y-tube olfactometer when the *S. endius* were provided a choice of *Z. cucurbitae* pupae in (1) acetone-treated versus distilled-water-treated odour source bottles; (2) acetone-treated versus abamectin-treated odour source bottles; and (3) distilled-water-treated versus abamectin-treated odour source bottles. The abamectin standard was diluted in acetone (12 mg a.i./mg). Four millilitres of acetone, diluted abamectin solution, or distilled water were pipetted into odour source bottles and rolled rapidly to ensure that the solution uniformly covered the inner wall of the bottle. After each type of solution had evaporated, each odour source bottle was filled with 200 three-day-old *Z. cucurbitae* pupae. For each test, more than 100 pairs of newly emerged *S. endius* adults were collected in a glass tube (4.5 cm in diameter, 11.5 cm in height) and provided with cotton balls saturated with a 10% honey solution for feeding. After one day, 100 females of *S. endius* were selected for testing one by one.

To investigate whether pre-exposure of *S. endius* to abamectin could eliminate the influence of abamectin odour on its host-selection behaviour, 500 *S. endius* pupae that developed into adults within two days were collected and placed into a glass tube (4.5 cm in diameter, 11.5 cm in height) which was treated with 12 mg a.i./kg of abamectin in advance. Similarly, equal quantities of *S. endius* pupae of the same age were separately collected into the two tubes—one treated with acetone and one with distilled water. After three days, 100 female *S. endius* adults were separately selected from each tube for use in behavioural tests. The behavioural responses of *S. endius* were examined by providing them with a choice of *Z. cucurbitae* pupae in (1) acetone-treated versus distilled-water-treated odour source bottles; (2) abamectin-treated versus distilled-water-treated odour source bottles; and (3) abamectin-treated versus acetone-treated odour source bottles.

For each test, a single *S. endius* was released at the entrance of the main Y-tube. In the olfactometer, walking or lingering by the test parasitoid beyond half of either arm of the Y-tube for a minimum of 20 s was recorded as a response. Test parasitoids that did not make a choice within 10 min were recorded as ‘no response’. Each parasitoid was tested only once. To eliminate the influence of spatial asymmetry of the Y-tube olfactometer or the *S. endius* responding to different room cues available from the right versus left arm of the tuber, the odour sources were switched after testing 10 parasitoids. After testing 10 parasitoids, the Y-tube was cleaned with alcohol (70% v) and distilled water and then dried in a drying oven at 120 °C for 20 min.

### 2.6. Control Efficacy of the Combined Application of S. endius and Abamectin Against B. cucurbitae Under Simulated Indoor Conditions with Different Application Parameters

We evaluated the optimum combination of *S. endius* and abamectin and the effect of their application sequence on the control efficacy against *Z. cucurbitae*. A commercial insecticide, abamectin 1.8% microemulsion, was used for testing after dilution with distilled water to a concentration of 12 mg a.i./kg. Prior to the test, 500 *S. endius* pupae that developed into adults within two days were collected and placed into a glass tube (4.5 cm in diameter, 11.5 cm in height) treated with the abamectin solution in advance. After three days, the live *S. endius* individuals were selected for testing. At the same time, a batch of soil was collected from the bitter gourd field at Qiufan Experiment Station of the Chinese Academy of Tropical Agricultural Sciences, Danzhou, China, and dried in a drying oven at 105 °C to a constant weight. The soils were then screened using a sieve (1.4 mm pore diameter) and transferred into 30 plastic boxes (35 cm × 24 cm × 14 cm). Each box contained 3 kg of soil. These boxes were placed into 30 nylon cages (40 cm × 40 cm × 40 cm, 0.6 mm pore diameter) to prevent the *Z. cucurbitae* from escaping but allowing *S. endius* to enter and exit.

All tests were evaluated in the above cages. Three treatments were set up to simulate the possible application sequence of *S. endius* and abamectin in field, as follows: (1) an amount of 20 mL of abamectin solution (TAS) was sprayed onto the soil surface, and then, 200 seven-day-old *Z. cucurbitae* larvae (TZL) were placed into the box to pupate. A total of 20 pairs of adult *S. endius* (TSE) were released into the cage after one day; (2) TZL was placed in the box and TSE released into the cage after one day. TAS was sprayed onto the soil surface after five days; and (3) TZL was placed into the box, and TAS was sprayed onto the soil surface after one day. Subsequently, TSE was released into the cage two hours later. Two controls were set up to exclude the influence of natural mortality of *Z. cucurbitae* on each treatment; (1) 20 mL of distilled water (TDW) was sprayed onto the soil surface, and then, TZL was placed into the box (matching the first treatment); (2) TZL was placed in the box to pupate, and TDW was sprayed onto the soil surface after six days (matching the second and third treatments). All solution was sprayed using a high-pressure sprayer (Yinghan Horticulture Co., Ltd., Zhongshan, China)). Each treatment and control were repeated six times. The number of adult *Z. cucurbitae* was recorded in each treatment and control cage after seven days.

### 2.7. Control Efficacy of Spalangia endius in Combination with Abamectin Against Bactrocera cucurbitae in the Field

Two bitter gourd fields (approximately 2400 m^2^) at the Qiufan Experimental Station of the Chinese Academy of Tropical Agricultural Sciences, Danzhou, China, were selected as experimental sites that were not sprayed with insecticides or fungicides during the experimental period. The two fields were located 2 km apart. Before testing, a batch of nylon cages (40 cm × 40 cm × 50 cm, 0.6 mm pore diameter) were buried in the bitter gourd field at a depth of 10 cm. Each cage had an open bottom and a 20 cm long zipper opening on one side. Commercial abamectin was diluted in distilled water to a concentration of 12 mg a.i./kg. Prior to the test, more than 1000 *S. endius* pupae that developed into adult parasitoids within two days were equally divided and placed into two glass tubes (4.5 cm in diameter, 11.5 cm in height). Each tube was treated with the abamectin solution in advance according to the method described in Section 2.5. After one day, live *S. endius* individuals were selected for testing.

Field trials were conducted on sunny days and at a temperature range of 21–31 °C. The tests were divided into three groups and conducted in nylon cages in one of the fields. For the combined application of *S. endius* and abamectin, 40 mL of abamectin solution (FAS) was sprayed on the soil surface, and then, TZL was introduced. After one day, TZE was released into the cage, and a cotton ball soaked in a 10% honey solution was hung in the centre. For the abamectin treatment group, FAS was sprayed on the soil surface, and then, TZL was introduced. For the *S. endius* treatment group, TZL was placed into the cage, and TSE was released after one day. For the host mortality control, to exclude the natural mortality of *Z. cucurbitae*, only TZL was placed in the cage in another bitter gourd field. All treatments were repeated 10 times. After seven days, the eclosion number of *Z. cucurbitae* was recorded in each cage.

### 2.8. Statistics

The toxicities of different insecticides to *S. endius* were evaluated using probit regression. Mortality was the dependent variable, and concentrations were the independent variables. The median lethal concentration (LC_50_) was fitted with a 95% confidence interval. Mortality and corrected mortality of *S. endius* were computed according to the method described by Abbott [19]. The parasitism rate of *S. endius* was calculated as the number of parasitised pupae divided by the total number of pupae. The percentage of host deaths was calculated by subtracting the natural mortality of *Z. cucurbitae* across the control from the total mortality of *Z. cucurbitae* in the respective treatment. The effects of the concentration and time since deposition of abamectin and the impact of the test method on the survival of *S. endius* were determined using a two-factor analysis of variance (two-way ANOVA), Tukey’s HSD test, and an independent samples *t*-test. Prior to conducting the ANOVA, the data, which included mortality and parasitism rates, were transformed using the arcsine square root transformation formula (arcsin√P).

The survival and parasitic efficacy of *S. endius* fed a combination of honey solution and abamectin, and the effect of the application sequence of abamectin on the combined control efficacy of *S. endius* and abamectin were determined using a one-way ANOVA combined with Tukey’s HSD test and a *t*-test. Before performing the ANOVA, the dataset, encompassing mortality and parasitism rates, underwent transformation using the formula arcsin√P. The data obtained from the Y-tube olfactometer were compared using the chi-squared test (χ^2^ test). The control efficacy of *S. endius* and abamectin on pupal *Z. cucurbitae* in the field was determined using nonparametric tests of independent samples, specifically by using the Kruskal–Wallis test. Differences at a probability level of *p* < 0.05 were considered significant. All statistical tests were performed using SPSS 20.0 for Windows (SPSS Inc., Chicago, IL, USA). Curve fitting was achieved using Origin 8.0 (Origin Lab Co., Northampton, MA, USA).

## 3. Results

### 3.1. Toxicities of Different Insecticides Against S. endius

According to the median lethal concentrations of the different insecticides, abamectin showed the least toxicity to *S. endius*, with LC_50_ of approximately 100 mg/L (Table 1). The LC_50_ of thiamethoxam was about half that value. Nitenpyram, emamectin, and beta-cypermethrin were highly toxic at concentrations of approximately 10 mg/L or less.

### 3.2. Effect of Concentration and Time Since Deposition of Abamectin and of the Test Method on the Survival of S. endius

The concentration (*F* = 32.13; df = 3, 96; *p* < 0.01) and time since deposition (*F* = 32.13; df = 3, 96; *p* < 0.01) of abamectin significantly affected the survival of *S. endius* (Table 2). The interactions between concentration and deposition time (*F* = 9.30; df = 9, 96; *p* < 0.01) also showed a significant effect. No significant differences were observed between the two methods (*F* = 0.19; df = 1, 96; *p* = 0.66). The interactions between concentration and tested method (*F* = 0.20; df = 3, 96; *p* = 0.90); the interactions between time since deposition and tested methods (*F* = 0.24; df = 3, 96; *p* = 0.87); and the interactions between concentration, time since deposition, and tested method (*F* = 0.53; df = 9, 96; *p* = 0.85) did not have a significant effect. Further analysis indicated that there were no significant differences between the mortality of *S. endius* in tubes treated with 12 mg/kg or 15 mg/kg of abamectin and those treated in control tubes. When the tubes treated with 18 mg/kg of abamectin were deposited for four days, abamectin showed no obvious influence on the survival of *S. endius*.

### 3.3. Survival and Parasitism Efficacy of S. endius Fed with the Combination of Honey Solution and Abamectin

**Figure 1:**
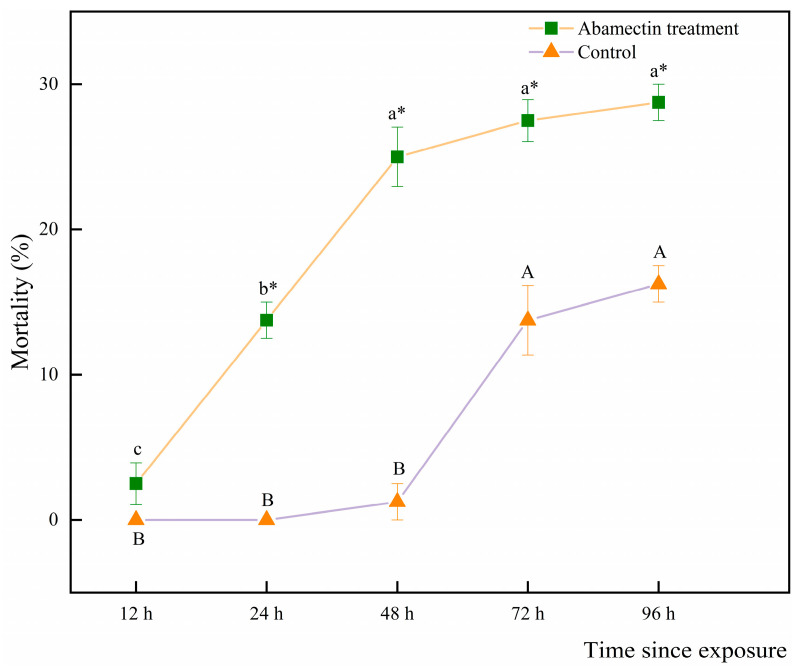
Mortality (mean ± standard error) of *Spalangia endius* fed the honey solution with or without abamectin treatment. Different and same upper- and lower-case letters represent significant differences in the control and treatment, respectively. Asterisks (*) indicate significant differences between the treatment and control groups (*t*-test; α = 0.05).

*Spalangia endius* fed a honey solution or a mixture of honey solution and abamectin presented mortality (Figure 1). However, the mortality of *S. endius* fed the mixture of honey solution and abamectin was significantly higher than that of *S. endius* fed the honey solution only from the 24th hour (12 h: *t* = 1.73, df = 14, *p* = 0.13; 24 h: *t* = 11.00, df = 14, *p* < 0.01; 48 h: *t* = 9.92, df = 14, *p* < 0.01; 72 h: *t* = 4.92, df = 14, *p* < 0.01; 96 h: *t* = 7.07; df = 14; *p* < 0.01).

**Figure 2:**
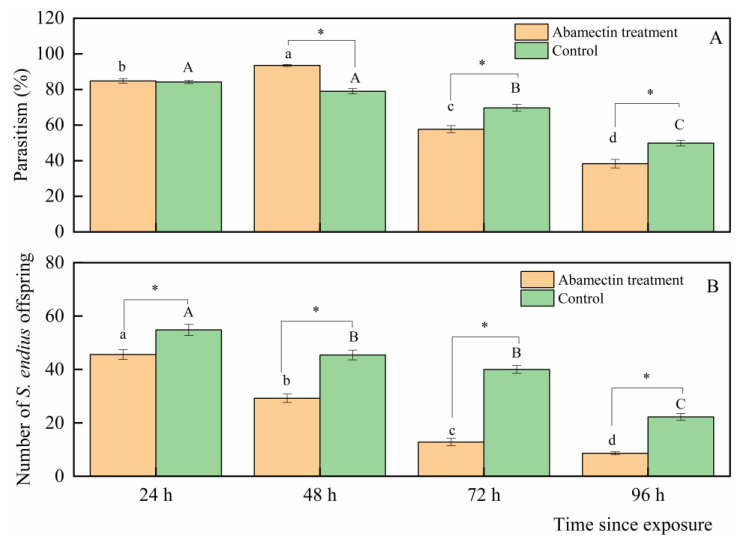
Parasitism (mean ± standard error) and fecundity (mean ± standard error) of 10 *Spalangia endius* females fed on the mixture of honey solution and abamectin in the four days following exposure. Figure (**A**,**B**) represent the parasitism and fecundity of *S. endius*, respectively. Means within the same lower-case and upper-case letters are not significantly different in the same treatment and control, respectively (Tukey’s HSD test; α = 0.05). Asterisks (*) indicate significant differences between the treatment and control groups (*t*-test; α = 0.05).

Parasitism by *S. endius* was affected when *S. endius* were fed a mixture of honey and abamectin solution (Figure 2A). Compared to the control, the parasitism levels of *S. endius* fed on the abamectin–honey solution increased significantly from the 48th hour (t = 9.71; df = 18; *p* < 0. 01); however, these levels decreased from the 72nd hour (*t* = −4.40; df = 18; *p* < 0.01) and 96th hour (*t* = −4.03; df = 18; *p* < 0.01). No significant differences were observed between the abamectin treatment and control at the 24th hour (*t* = 0.40; df = 18; *p* = 0.70).

The number of offspring of *S. endius* was also influenced by feeding on a mixture of honey and abamectin solution (Figure 2B). Compared to the control, the number of offspring of the mixture-fed *S. endius* clearly decreased at all tested times (12 h: *t* = −6.28, df = 18, *p* < 0.01; 24 h: *t* = −10.3, df = 18, *p* < 0.01; 48 h: *t* = 9.92, df = 18, *p* < 0.01; 72 h: *t* = −13.8, df = 18, *p* < 0.01; 96 h: *t* = −9.65; df = 18; *p* < 0.01).

### 3.4. Effect of Abamectin on the Host-Selection Behaviour of S. endius

**Figure 3:**
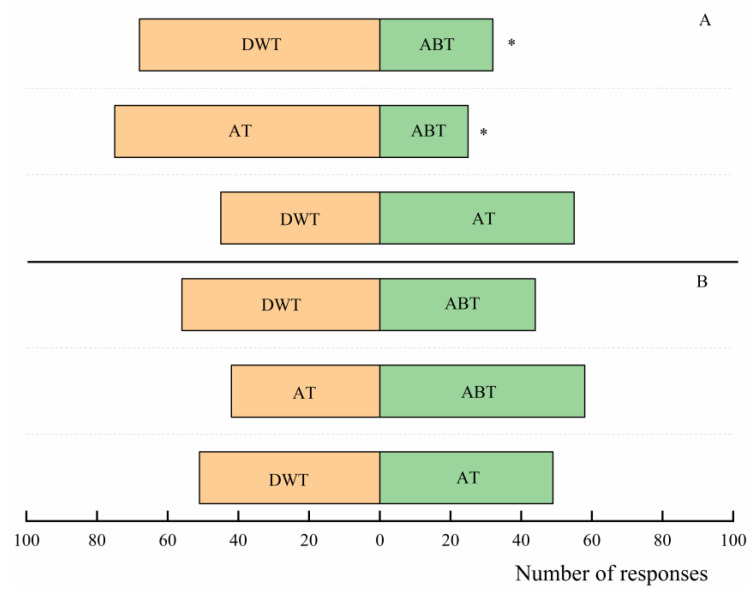
Selection behaviour of *Spalangia endius* towards *Zeugodacus cucurbitae* pupae in different odour source bottles. Figures (**A**,**B**) show the selection behaviour of *S. endius* without and with abamectin experience, respectively. AT, DWT, and ABT represent *Z. cucurbitae* pupae in acetone-treated, distilled water-treated, and abamectin-treated odour source bottles, respectively. Asterisks (*) indicate significant differences between the two datasets (χ^2^ test; α =0.05).

Abamectin significantly affected the selection behaviour of *S. endius* towards *Z. cucurbitae* pupae (Figure 3A). *Splangia endius* showed obvious avoidance towards the pupae from the odour source bottle treated with abamectin compared to the pupae from the odour source bottles treated with distilled water or acetone (water: χ^2^ = 12.96; df = 1; *p* < 0.01; acetone: χ^2^ = 25.00; df = 1; *p* < 0.01). However, *S. endius* showed no significant preference towards the pupae from the odour source bottles treated with distilled water or acetone (χ^2^ = 1.00; df = 1; *p* = 0.32).

When the *S. endius* pupae that developed into the adult parasitoids within two days were exposed to abamectin in advance, *S. endius* showed no significant avoidance against the pupae from the odour source bottle treated with abamectin compared to the pupae in the acetone-treated (Figure 3B: χ^2^ = 2.56; df = 1; *p* = 0.11) or distilled water-treated bottles (χ^2^ = 1.44; df = 1; *p* = 0.23). In addition, *S. endius* showed no significant preference towards the pupae from the odour source bottles treated with distilled water or acetone (χ^2^ = 0.04; df = 1; *p* = 0.84).

### 3.5. Effect of the Application Sequence of Abamectin on the Combined Control Efficacy of S. endius and Abamectin

**Figure 4:**
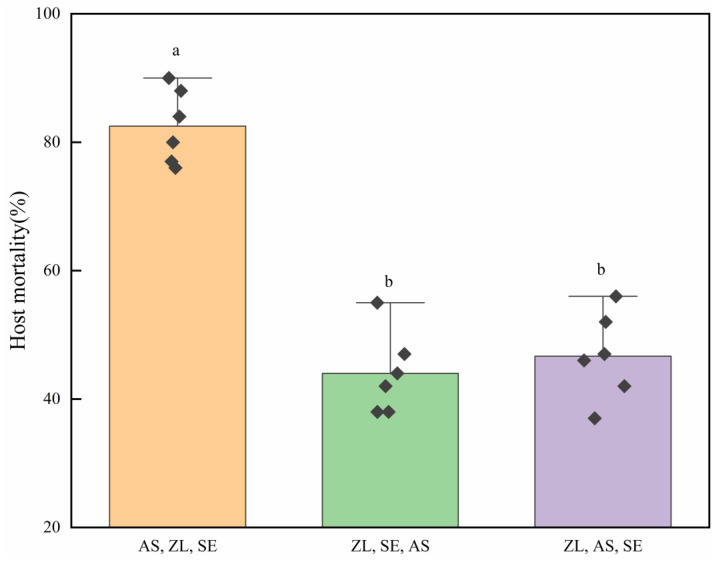
Effect of treatment combination sequence on the percentage host mortality (mean ± standard error). ‘AS, ZL, SE’ represents spraying abamectin initially, followed by releasing pupae and then parasitoids sequentially; ‘ZL, SE, AS’ involves releasing pupae and parasitoids first and then applying abamectin; and ‘ZL, AS, SE’ consists of introducing pupae first and subsequently applying abamectin and parasitoids in sequence, respectively. Means within the same lower-case letters are not significantly different (Tukey HSD test; α = 0.05).

The sequence of abamectin application significantly affected the combined control efficacy of *S. endius* and abamectin (Figure 4, *F* = 40.79; df = 2; *p* < 0.01). When abamectin was utilised before the host pupated, the combined control efficacy of *S. endius* and abamectin was obviously higher than that of the association in which abamectin was utilised after the host pupated. The results also showed that the sequence of application of abamectin did not significantly influence the combined control efficacy of *S. endius* and abamectin when the latter was utilised after the host pupated (*p* > 0.05).

### 3.6. Control Efficacy of S. endius and Abamectin Alone or Together in Field

**Figure 5:**
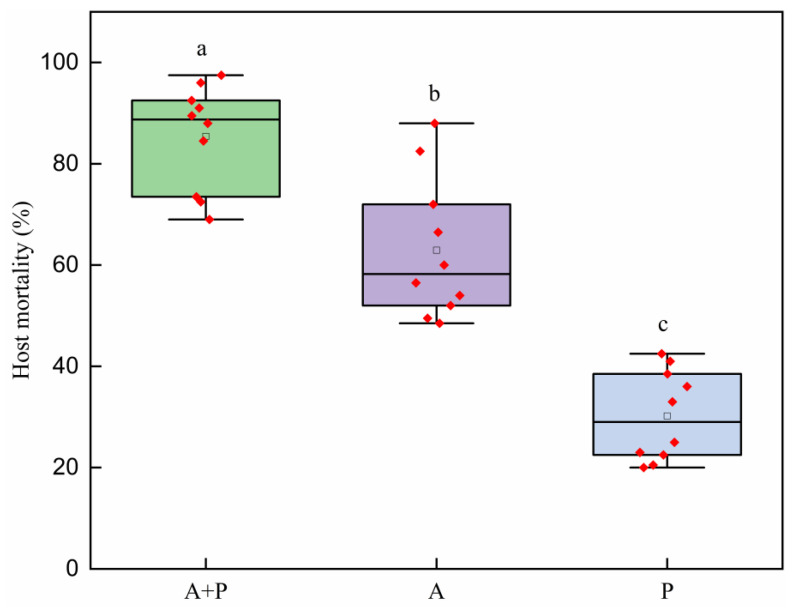
Control efficacy (mean ± standard error) of *Spalangia endius*, abamectin, and their combinations on the pupal *Zeugodacus cucurbitae* in field. A, P, and A + P represent the application of abamectin, *S. endius*, and their combination, respectively. Means within the same lower-case letters are not significantly different (Kruskal–Wallis test; α = 0.05).

The control efficacy of *S. endius* and abamectin alone or together significantly differed (Figure 5: *H* = 23.81; df = 2; *p* < 0.01). The combined application of abamectin and *S. endius* resulted in a higher mortality of *Z. cucurbitae* than their separate application (abamectin: adjusted *p* = 0.04; parasitoid: adjusted *p* < 0.01). The results also indicated that abamectin had a higher control efficacy against *Z. cucurbitae* than *S. endius* (adjusted *p* = 0.02).

## 4. Discussion

The parasitoid wasp *Eretmocerus hayati*, a dominant natural enemy of the notorious agricultural pest *Bemisia tabaci*, exhibits strong compatibility with abamectin. Studies have demonstrated that while abamectin application prolongs the pupal development period of *E. hayati*, it does not significantly impair its parasitism ability and fecundity [20]. In the present study, abamectin was also a suitable insecticide for combination with *S. endius*. However, some studies have demonstrated that abamectin is not suitable for combined application with parasitoids. For instance, abamectin exhibits high acute toxicity towards *Encarsia formosa*, a parasitoid of *B. tabaci* [21]. Abamectin also shows high toxicity towards *Neochrysocharis formosa* and *Ganaspidium nigrimanus*, significant parasitoids of *Liriomyza trifolii* [22]. This indicates that different parasitoid species vary in their sensitivity to abamectin. In addition, the current study found that abamectin had the least contact toxicity towards *S. endius*. However, when the parasitoids ingested the honey solution containing abamectin, their survival rate, parasitism rate, and offspring number were all affected to a certain extent. This suggests that the impact of abamectin on parasitoids is also influenced by its application method. Interestingly, 48 h after ingesting the honey solution containing abamectin, the parasitism rate of *S. endius* on the pupae of *Z. cucurbitae* was significantly higher than that of those only ingesting the honey solution. Analogous to this research, the parasitoid *E. formosa*, upon contact with residues of nitenpyram, imidacloprid, thiamethoxam, cyantraniliprole, sulfoxaflor, and acetamiprid, also exhibited a significantly higher rate of host parasitism five days after treatment compared to the control that did not encounter any insecticides [23]. These findings indicate that parasitoids are likely to display hormetic effects within a specific period after being stressed by insecticides.

Numerous studies have demonstrated that the effects of insecticides on parasitoids extend beyond their direct toxicity. Many insecticides emit odours that negatively impact the olfactory behaviour of parasitoids. For instance, *Bacillus thuringiensis* Berliner exhibits a strong repellent effect on the lepidopteran parasitoid *Palmistichus elaeisis* [24]. Similarly, the odours of esfenvalerate, methamidophos, and vanilla can disrupt the food-searching behaviour of *Microplitis croceipes* [25]. Furthermore, acetamiprid, dimethoate, flupyradifurone, and sulfoxaflor have been shown to impair the host-searching ability of *Nasonia vitripennis* [26]. In the current study, *S. endius* showed obvious avoidance towards the pupae from the odour source bottle treated with abamectin compared to the pupae from the odour source bottles treated with distilled water or acetone. This indicates that the odour of abamectin affects the host-selection behaviour of *S. endius*. Shi et al. demonstrated that imidacloprid exposure significantly disrupts the host-searching behaviour of the fruit fly parasitoid *Leptopilina drosophilae*. Subsequent transcriptomic and functional analysis revealed that this behavioural alteration is associated with the downregulation of certain chemoreception genes [27]. Subsequent investigations could reveal the molecular mechanism underlying abamectin’s disruption of host-searching behaviour in *S. endius* by characterizing its regulatory effects on key olfactory gene families. Previous studies have indicated that the learning and experience of generalist parasitoids can change their selection behaviour toward hosts [28,29,30]. Rafalimanana et al. demonstrated that chlorpyrifos could affect the host-searching and host-locating behaviour of the parasitoid *Leptopilina heterotoma*. However, after short-term olfactory conditioning, the parasitoid that had been pre-exposed to chlorpyrifos could locate hosts more rapidly than those that had not been exposed to chlorpyrifos [31]. In the current study, when the emerging *S. endius* were placed into the glass tube treated with abamectin, abamectin did not have an obvious influence on their odour-based host-selection behaviour. This may be attributed to the ability of short-term olfactory conditioning to counteract the adverse effects of abamectin on the olfactory behaviours of *S. endius*.

Previous studies have demonstrated that the LC_50_ of abamectin against *Z. cucurbitae* adults, mature larvae (corresponding to seven-day-old larvae), and pupae were 18.66, 1.88, and 129.36 mg/L, respectively [32]. This indicates that abamectin exhibits the highest toxicity towards mature larvae compared to the other two developmental stages. In the present study, the three combinations tested were as follows: (1) spraying abamectin first, followed by releasing the hosts and then the parasitoids; (2) placing the hosts and then the parasitoids, and finally, applying abamectin; and (3) placing the hosts, followed by abamectin, and then the parasitoids. These combinations resulted in 82.25%, 43.92%, and 47.17% host mortality, respectively. This is likely because abamectin is more toxic to the mature larvae of *Z. cucurbitae* than to the pupae. In the field, the combination of abamectin and *S. endius* resulted in 85.40% host mortality. However, combined with the natural mortality of *Z. cucurbitae* pupae, the mortality could reach 100%. These findings also indicate that the pupation time and peak of *Z. cucurbitae* should be monitored to determine the key period for the most effective combined utilisation of abamectin and *S. endius.*

In conclusion, abamectin can negatively influence survival and fecundity of *S. endius* by ingestion if abamectin is provided in a substrate that the parasitoid is likely to eat. However, if 12 mg a.i./kg of abamectin is applied on the soil containing mature host larvae, and adult *S. endius* are released only after host pupation has occurred, abamectin can be effectively used in combination with *S. endius*. This application sequence is the most effective.

## Figures and Tables

**Table 1:** Toxicity of different insecticides against *Spalangia endius*.

Insecticide Types	Regression Equations	LC_50_ (mg/L)	95% Confidence Intervals
Abamectin	y= (−3.89 ± 0.45) + (1.93 ± 0.26)X	104.42	83.03–147.82
Emamectin benzoate	y= (−0.51 ± 0.08) + (0.55 ± 0.09)X	8.32	4.79–17.63
Thiamethoxam	y= (−1.89 ± 0.25) + (1.09 ± 0.15)X	54.75	41.59–73.67
Nitenpyram	y= (−0.63 ± 0.13) + (0.63 ± 0.12)X	10.20	6.33–17.34
Beta cypermethrin	y= (−0.68 ± 0.23) + (2.48 ± 0.25)X	1.88	0.14–5.09

**Table 2:** Effect of time since deposition and abamectin test method on the survival of *Spalangia endius* (mean ± standard error).

Concentrations (mg/kg)	Deposition Time	Residual Film Method		Impregnated Filter Method	
		Mortality (%)	Corrected Mortality (%)	Mortality (%)	Corrected Mortality (%)
12	0 d	1.25 ± 1.25 a	1.25	2.50 ± 2.50 a	1.27
	2 d	1.25 ± 1.25 a	0.00	1.25 ± 1.25 a	1.25
	4 d	0.00 ± 0.00 a	0.00	0.00 ± 0.00 a	0.00
	6 d	0.00 ± 0.00 a	0.00	0.00 ± 0.00 a	0.00
15	0 d	3.75 ± 2.39 a	3.75	2.50 ± 1.44 a	1.27
	2 d	1.25 ± 1.25 a	0.00	0.00 ± 0.00 a	0.00
	4 d	0.00 ± 0.00 a	0.00	0.00 ± 0.00 a	0.00
	6 d	0.00 ± 0.00 a	0.00	0.00 ± 0.00 a	0.00
18	0 d	11.25 ± 1.25 a*	11.25	10.00 ± 0.00 a	8.86
	2 d	8.75 ± 1.25 a*	7.59	11.25 ± 1.25 a	11.25
	4 d	1.25 ± 1.25 b	1.25	0.00 ± 0.00 b	0.00
	6 d	0.00 ± 0.00 b	0.00	0.00 ± 0.00 b	0.00

The control mortality of *S. endius* was 1.25 ± 1.25. Means within a row followed by the same lower-case letters are not significantly different in the same treatment (Tukey’s HSD test; α = 0.05). Asterisk (*) indicates significant differences between the treatment and control groups (*t*-test; α = 0.05).

## Data Availability

The original contributions presented in this study are included in the article. Further inquiries can be directed to the corresponding authors.
